# Novel high-grade serous epithelial ovarian cancer cell lines that reflect the molecular diversity of both the sporadic and hereditary disease

**DOI:** 10.18632/genesandcancer.76

**Published:** 2015-09

**Authors:** Hubert Fleury, Laudine Communal, Euridice Carmona, Lise Portelance, Suzanna L. Arcand, Kurosh Rahimi, Patricia N. Tonin, Diane Provencher, Anne-Marie Mes-Masson

**Affiliations:** ^1^ Centre de recherche du Centre hospitalier de l'Université de Montréal (CRCHUM), Montreal, Canada; ^2^ Institut du cancer de Montréal, Montreal, Canada; ^3^ The Research Institute of the McGill University Health Centre, Montreal, Canada; ^4^ Department of Pathology, Centre hospitalier de l'Université de Montréal (CHUM), Montreal, Canada; ^5^ Department of Human Genetics, McGill University, Montreal, Canada; ^6^ Department of Medicine, McGill University, Montreal, Canada; ^7^ Division of Gynecologic Oncology, Université de Montréal, Montreal, Canada; ^8^ Department of Medicine, Université de Montréal, Montreal, Canada

**Keywords:** cell lines, high-grade serous, BRCA mutations, epithelial ovarian cancer

## Abstract

Few cell line models of epithelial ovarian cancer (EOC) have been developed for the high-grade serous (HGS) subtype, which is the most common and lethal form of gynaecological cancer. Here we describe the establishment of six new EOC cell lines spontaneously derived from HGS tumors (TOV2978G, TOV3041G and TOV3291G) or ascites (OV866(2), OV4453 and OV4485). Exome sequencing revealed somatic *TP53* mutations in five of the cell lines. One cell line has a novel *BRCA1* splice-site mutation, and another, a recurrent *BRCA2* nonsense mutation, both of germline origin. The novel *BRCA1* mutation induced abnormal splicing, mRNA instability, resulting in the absence of BRCA1 protein. None of the cell lines harbor mutations in *KRAS* or *BRAF*, which are characteristic of other EOC subtypes. SNP arrays showed that all of the cell lines exhibited structural chromosomal abnormalities, copy number alterations and regions of loss of heterozygosity, consistent with those described for HGS. Four cell lines were able to produce 3D-spheroids, two exhibited anchorage-independent growth, and three (including the *BRCA1* and *BRCA2* mutated cell lines) formed tumors in SCID mice. These novel HGS EOC cell lines and their detailed characterization provide new research tools for investigating the most common and lethal form of EOC.

## INTRODUCTION

Ovarian cancer is the fifth cause of cancer-related deaths in the Western world, the second most common gynecological cancer and the leading cause of death from gynecological malignancies [1]. The most common form of cancer of the ovary is epithelial ovarian cancer (EOC) (reviewed in [2, 3]). Largely asymptomatic, over 70% of EOC patients are diagnosed at an advanced stage of the disease. Standard first line therapy consists of tumor de-bulking surgery and treatment with platinum DNA alkylating-based combination chemotherapy, such as carboplatin or cisplatin, associated with the microtubule poison paclitaxel [4, 5]. Initial response rates are high but probably due to innate or acquired chemoresistance the disease recurs in more than 70% of patients [5, 6]. EOCs can be subdivided in different subtypes according to their cell type: serous, mucinous, endometrioid, clear cell, mixed subtypes or undifferentiated adenocarcinomas or carcinomas [2, 7]. For the serous subtype, there is an understanding that distinct molecular events contribute to two possibly non-contiguous diseases, which differentiate low (LGS) and high grade serous (HGS) cancers. Furthermore, it is becoming increasingly clear that distinct molecular events are associated with each particular EOC subtype (discussed in [2, 7-9]).

As a group HGS tumors account for over 60% of all EOCs and are characterized by high genomic instability and chromosomal anomalies with cells exhibiting complex karyotypes, which include intrachromosomal breaks and aneuploidy [10, 11]. It has been proposed that mutations in the *TP53* tumor suppressor gene is one of earliest mutational events in HGS as nearly all cases harbor a somatic mutation in this gene as confirmed by a recent molecular genetic profiling of over 300 HGS samples by The Cancer Genome Atlas Research (TCGA) network [12, 13]. Furthermore, compromised homologous recombination due to loss of BRCA1 and BRCA2 function, by the inheritance of a germline pathogenic mutation in approximately 10-20% of HGS cases [12], or through somatic means by acquiring an intragenic mutation or epigenetic silencing [12, 14, 15], occurs in approximately one third of the HGS EOC cases. The prevalence of *TP53* mutations and *BRCA1*/*BRCA2* deficiency likely leads to incompetent DNA repair which in turn contributes to chromosomal instability, resulting in severely aberrant karyotypes. In general, HGS EOCs are highly heterogeneous having widespread inter- and intra-tumoral mutation profiles, although common patterns of chromosomal regions of gain and loss have been observed [12, 16-18].

The study of processes or pathways relevant to HGS EOC biology, treatment or etiology has been facilitated by cell-based models [19-22]. However, EOC cell lines representative of HGS based on their intragenic mutational spectrum, chromosomal anomalies and gene expression profiles are limited in number [23]. This is surprising given the high frequency of HGS cases relative to the other subtypes of EOC. Indeed the most commonly used cell lines, such as SKOV3 do not exhibit features characteristic of HGS as shown in a recent molecular genetic analysis of 47 ovarian cancer cell line models [23]. It has also been reported that several tumor cell banks lack information of tumor of origin, grade, stage and molecular marker characterization for several ovarian cancer cell lines [24]. Moreover, only a few HGS EOC cell lines harboring *BRCA1* or *BRCA2* mutations have been described [23, 25, 26]. Recently, we reported the characterization of a set of nine matched EOC cell lines derived from tumors at diagnosis and recurrence, and from ascites from each of three serous EOC patients [27], and they have been used to characterize some of the molecular genetic features of HGS. In all, it is clear that cell line models that accurately recapitulate HGS EOC characteristics and heterogeneity are in urgent need as they are a starting point to discover and validate new therapeutic targets and biomarkers of this disease.

Here we have described the generation, molecular characterization, and *in vivo* and *in vitro* growth characteristics of six new EOC cell lines all derived from HGS cases. Two of these cell lines were derived from patients harboring a germline *BRCA1* or *BRCA2* mutation. The molecular genetic characterizations of these cell lines suggest that they exhibit features consistent with HGS tumors. Their unique profiles, which include different mechanisms of inactivation of *TP53* and *BRCA1*, reflect the heterogeneity observed in HGS tumors. Therefore, these new cell lines offer a significant contribution to the list of HGS EOC cell lines available for research as they present distinct features characteristic of this multiple-faceted disease.

## RESULTS

### Cell lines derived from HGS EOC patient samples

The clinical characteristics of the patients 866, 2978, 3041, 3291, 4453, and 4485 from which the cell lines were derived are shown in Table 1. All patients had late stage (III-IV) and high-grade serous disease. Patients had complete, sub-optimal or optimal surgical debulking, received standard first-line platinum/taxol chemotherapy treatment and died from disease progression.

**Table 1:** Clinical features of patients and tumor characteristics of samples used to derive cell lines

Patient Identification	866	2978	3041	3291	4453	4485
Age of diagnosis (years)	60	63	61	59	70	55
Survival (months)	10	27	24	11	9	26
BRCA hereditary status	negative	negative	negative	negative	BRCA2(+)	BRCA1(+)
Histopathology sub-type	high-grade serous	high-grade serous	high-grade serous	high-grade serous	high-grade serous	high-grade serous
Disease stage	IIIC	IIIC	IVA	IIIC	IIIC	IIIC
CA125 (U/ml) at presentation	234	2397	2179	1631	2808	593
Cytoreduction	sub-optimal	sub-optimal	sub-optimal	optimal	complete	optimal
First-line treatment	1. surgery 2. carboplatin/taxol (iv)	1. surgery 2. carboplatin/taxol (iv)	1. carboplatin/taxol (iv) 2. surgery 3. cisplatin/taxol (ip,iv)	1. surgery 2. cisplatin/taxol (ip,iv)	1. carboplatin/taxol (iv) 2. interval surgery	1. carboplatin/taxol (iv) 2. interval surgery 3. maintenance sorafenib vs placebo trial
Platinum response (6 months after first-line)	disease progression*	disease progression	progression-partial response	disease progression	disease progression	disease progression
Progression as cause of death	Yes	Yes	Yes	Yes + mesenteric ischemia	Yes	Yes
Previous personal history of cancer	No	No	No	No	breast cancer	No
Prior oncologic treatment	No	No	breast cancer prevention trial-tamoxifen	No	radiotherapy & hormono-therapy	No
Year of sampling	2000	2006	2007	2007	2009	2011
Cell line designation	OV866(2)	TOV2978G	TOV3041G	TOV3291G	OV4453	OV4485
Chemotherapy naïve at sample collection	No	Yes	No	Yes	Yes**	No

* Disease progression was defined as increased levels of blood CA125 and tumor size assessed by imaging.

** Note that this patient had received radiotherapy and hormonotherapy treatment for breast cancer five years prior to ovarian cancer diagnosis, but ovarian tumor samples were collected before any chemotherapy treatment for ovarian cancer.

The cell lines were derived from samples collected at diagnosis or at the time of relapse, from either solid tissue (TOV) or ascites (OV) (Table 1). The TOV2978G, TOV3291G and OV4453 cell lines were derived from primary tissues or ascites from patients that had not had chemotherapy prior to surgery; whereas the OV866(2), TOV3041G and OV4485 cell lines were derived from samples obtained from recurring disease and thus the patients had been treated with chemotherapy before collection. The OV866(2) cell line nomenclature reflects the fact that it was established from a second ascites sample obtained from patient 866. The OV4453 cell line was derived from a patient who received radiotherapy and hormonotherapy treatments for breast cancer five years prior to ovarian cancer diagnosis. Among these six cell lines, OV4453 is the only example of a cell line that was derived from a patient with a personal prior history of cancer.

**Figure 1:**
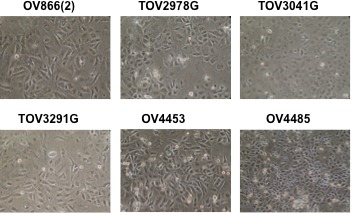
Morphology of cell lines derived from patients 866, 2978, 3041, 3291, 4453 and 4485 Shown are light microscopy images of cell lines at 80% confluence at passages between 66 and 73. Cell lines had developed into predominantly small epithelial type cells, often aggregated, and fibroblast shaped cells were notably absent.

After 50 passages, the cell lines appeared homogeneous in their morphology, exhibiting a cobblestone appearance characteristic of epithelial cells (Figure 1). Notable is the scarcity or lack of elongated fibroblast-like cells in culture.

### Cell lines derived from BRCA1 and BRCA2 mutation positive cases

It is well established that germline mutations in *BRCA1* and *BRCA2* confer susceptibility to breast and ovarian cancers. *BRCA2* and *BRCA1* germline mutations were identified in patients 4453 and 4485, respectively, as a consequence of genetic testing in hereditary cancer clinics due to their breast and ovarian cancer family history (see Figure supplemental S1 for pedigree). The *BRCA2*:G6085T (c.5857G>T) mutation was identified in a screen of the most common *BRCA1* and *BRCA2* mutations found to recur in French Canadians due to common ancestry [28]. Indeed, all of the cell lines were derived from patients who self reported French Canadian ancestry, a population known to exhibit strong founder effects [29]. This *BRCA2* mutation was found in the homozygous state in OV4453 cell line (Table 2) based on DNA sequencing and SNP array analyses (see below). This variant introduces a stop codon (p.E1953X) in the amino acid sequence that is predicted to result in a truncated BRCA2 protein. This mutation is one of the most common *BRCA2* deleterious mutations found in French Canadians [28, 29].

**Table 2:** Genetic and copy number alteration results of candidate loci in EOC cell lines

			Cell line					
Platform	Feature	Gene	OV866(2)	TOV2978G	TOV3041G	TOV3291G	OV4453	OV4485
Whole exome sequencing and targeted mutation analyses	Mutations in HGS	TP53	c.745 A>T (R249W)	c.920-2 A>G (splice)	N.I.	c.745 A>T (R249W)	c.376-1 G>A (splice)	c.818 G>A (R273H)
		BRCA1	N.I.	N.I.	N.I.	N.I.	N.I.	c.4485-1 G>T (splice)
		BRCA2	N.I.	N.I.	N.I.	N.I.	c.5857 G>T (E1953X)	N.I.
		CSMD3	N.I.	N.I.	N.I.	N.I.	c.1937 G>C (S646T)	N.I.
		NF1	N.I.	N.I.	N.I.	N.I.	N.I.	N.I.
		CDK12	N.I.	N.I.	N.I.	c.3095+1 G>A (splice)	N.I.	N.I.
		RB1	N.I.	N.I.	N.I.	N.I.	c.2490-5_ 2490-1 del5 (splice)	N.I.
	Mutations in Non-HGS	KRAS, BRAF, ARID1A, PIK3CA, CTNNB1	N.I.	N.I.	N.I.	N.I.	N.I.	N.I.
SNP array analyses	CNA^gain^ in HGS	MYC	5	7	6	6	4	2
		MECOM	5	4	6	9//8	3	3
		CCNE1	9	5	4	8	4	2
		KRAS	14	2	5	7	4	2
		ALG8	3	4	4	4	3	2
		SOX17	4	6	3	5	3	2
		TACC3	3	5	5	3	3	2
	CNA^loss^ in HGS	NF1	3//2	2	2	4	2	1
		RB1	3	2	2	3	2	1
		PTEN	3	3	3	5	2	1
	Ploidy		3.62	3.82	4.07	5.31	2.69	1.62
	Number of segments		535	541	470	637	502	128

Targeted mutation analyses were performed for BRCA1, BRCA2, TP53, BRAF and KRAS. No predicted deleterious mutation identified indicated by N.I. Copy number for overlapping ASCAT segment shown for each gene locus. Change in copy number in gene locus indicated by //. Copy number gain (dark gray highlighted) or loss (light gray highlighted) is shown relative to ploidy only for each category (CNA^gain^ or CNA^loss^).

**Figure 2:**
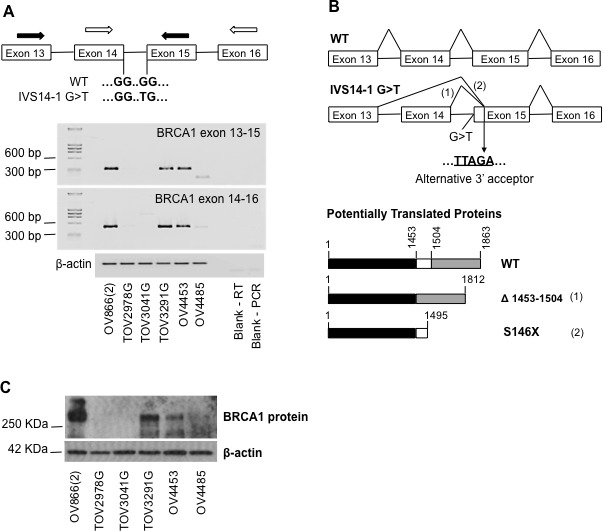
Characterization of the new BRCA1 intronic mutation identified in the OV4485 cell line A) Primer design for amplification of *BRCA1* cDNA surrounding the intronic mutation (black arrows, exon 13-15; white arrows, exon 14-16), and agarose gel of obtained PCR products. PCR amplification of β–actin was used as a control. B) Schematic illustration of alternative *BRCA1* mRNA splicing, alternative 3′acceptor of exon 15, and predicted translated BRCA proteins obtained resultant from cDNA sequencing of the OV4485 PCR products. C) Detection of BRCA1 protein in cell lysates of the different cell lines by Western blot. β–actin was used as loading control.

Following mutation negative findings due to a targeted mutation screen of the most common *BRCA1*/*BRCA2* mutations found in French Canadians, a comprehensive commercial-based mutation analysis performed in the hereditary cancer clinic revealed that patient 4485 harbored the *BRCA1*:IVS14-1G>T (c.4485-1 G>T) mutation. This mutation was also found in OV4485 cell line (Table 2) and was found in the homozygous state based on DNA sequencing and SNP array analyses (see below). The pathogenic effect of this new *BRCA1*:IVS14-1G>T mutation is unknown. This unique variant has not been reported in either of the Breast Information Core mutation database (research.nhgri.nih.gov/bic/) or the LOVD-Leiden Open Variation Database (chromium.liacs.nl/LOVD2/cancer/home.php?select_db=BRCA1), nor the Exome Aggregation Consortium (ExAC) database (exac.broadinstitute.org). However, an investigation of this mutation in the OV4485 cell line suggests that it probably results in the inactivation of BRCA1 protein function (Figure 2). A PCR-based assay of cDNA identified the presence of novel transcript in this cell line (Figure 2A) that when sequenced suggested that it was derived from the splicing of exon 13 to an alternative 3′ acceptor in exon 15 likely due to the presence of G>T substitution at the splice acceptor site (Figure 2B). When translated to protein, two predicted BRCA1 proteins could be produced, one having a deletion of 51 amino acids (p.del 1453-1504) (exon 14 splice out + 29bp deletion on exon 15) and another having a premature stop codon (p.S1496X) (29bp deletion of exon 15) (Figure 2B). The p.del 1453-1504 mutation would delete part of the coiled coil structure (important for interaction with PALB2, and indirectly BRCA2) and some of the ATM target phosphorylation sites of BRCA1 [30, 31]. The introduction of a stop codon at amino acid position 1496 would also predict a truncated protein with loss of the BRCA1 C-terminus (BRCT) domain (important for interaction with several phosphorylated proteins that are primarily involved in the DNA damage response) [30, 31]. Rare BRCA1 codon 1496 truncating mutations (BRCA1: p.Ser1496Ter) have been described and are classified as clinically important in the Breast Information Core mutation database (http://research.nhgri.nih.gov/bic/). However, it is not known if these predicted transcripts are translated into truncated proteins or are eliminated by other mechanisms such as nonsense-mediated decay. In the OV4485 cell line, no evident BRCA1 protein band was detected (Figure 2C), and this could be due to low levels of transcribed message (Figure 2A). These results are consistent with the possibility that *BRCA1*:IVS14-1G>T mutation is clinically relevant.

Using a targeted mutation screen, the *BRCA1* and *BRCA2* germline mutations found to recur in French Canadians were not found in normal blood lymphocyte DNA from the patients from which the TOV2978G, TOV3041G and TOV3291G cell line lines were derived. Germline mutations were also not found in a more limited mutation screen of normal blood lymphocyte DNA from the patient from which OV866(2) cell line was derived. These results are consistent with our findings from whole exome sequencing analysis, which also revealed no further evidence for *BRCA1*/*BRCA2* mutations in these cell lines (Table 2). During the course of analyzing BRCA1 gene and protein expression to characterize the consequences of the *BRCA1*:IVS14-1G>T mutation, we were not able to detect BRCA1 message or protein in TOV2978G and TOV3041G (Figure 2A and C). It is possible that in these cell lines *BRCA1* expression was affected by epigenetic silencing of the promoter, as has been described by the TCGA to have occurred in approximately 12% of 316 HGS EOC tumor samples [12].

### Cell lines exhibit somatic genetic and genomic features characteristic of HGS EOC

In our initial study of the EOC cell lines, we performed a targeted mutation analysis of selected genes as *TP53* mutations have been found in the majority of HGS EOC tumors and mutually exclusive mutations in *KRAS* and *BRAF* have been found in LGS EOC tumors [12, 32-34]. All cell lines with the exception of TOV3041G harbored an intragenic somatic *TP53* mutation (Table 2). All three missense *TP53* mutations are classified as deleterious based on the IARC TP53 mutation database (www-p53.iarc.fr), and the two splice *TP53* mutations are predicted to affect the translation of the encoded protein [18]. Using a targeted mutation analysis, no *KRAS* and *BRAF* mutations affecting common mutation sites in these genes were identified in the cell lines.

**Figure 3:**
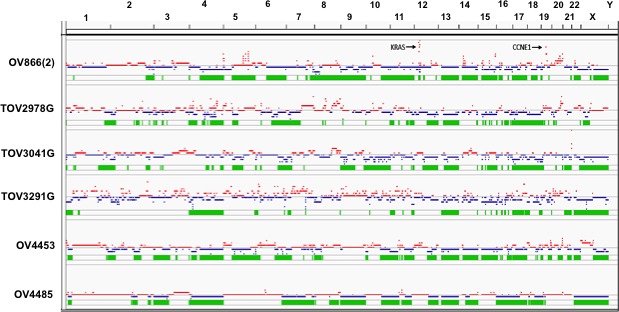
Genomic landscape of the HGS cell lines Total copy number of ASCAT derived segments was plotted for the genome on a scale of 0 to 15 copies using IGV. Segments with copy number above ploidy (see Table 2 for values) are indicated in red (CNV^gain^), and those below ploidy are blue (CNV^loss^). Regions of the genome with LOH are indicated in green and shown immediately below each CNA graph depicted for each cell line. Arrows indicate genomic locations containing amplification of *KRAS* and *CCNE1* loci in the genomic profile of the OV866(2) cell line.

SNP array genotyping analysis of each EOC cell line was performed to infer genome-wide chromosomal abnormalities as HGS tumors and cell lines exhibit a high frequency of chromosomal instability as displayed by copy number anomalies (CNA) across the genome [12, 23, 35]. SNP array analyses of these cell lines showed evidence of extensive chromosomal abnormalities consistent with intrachromosomal breaks and allelic imbalances, which are features characteristics of HGS tumors (Figure 3). Moreover, all cell lines exhibited loss of heterozygosity (LOH) across the *TP53* locus at 17p13, the *BRCA1* locus at 17q12, and the *BRCA2* locus at 13q13.

We also examined SNP array data for *MYC*, *MECOM*, *CCNE1*, *KRAS*, *ALG8*, *SOX17*, and *TACC3*, as regions containing these genes have been shown to exhibit amplification (in order of descending frequency) and for the *NFI*, *RB1* and *PTEN* loci as regions containing these genes have been shown to exhibit homozygous deletions (in order of descending frequency) in HGS tumors [12, 23]. CNA gain was observed for a number of loci in the EOC cell lines, where gain of the *MYC* and *MECOM* loci were observed in all of the EOC cell lines (Table 2). CNA gain was also distinctive for the *KRAS* and *CCNE1* loci in the OV866(2) cell line akin to classical gene amplification (Figure 3). Although CNA loss was observed for the *NFI*, *RB1* and *PTEN* loci in all of the EOC cell lines, there was no evidence for homozygous deletions for any of these genes.

We have performed whole exome sequencing analysis to evaluate the presence of somatic anomalies in *TP53*, *BRCA1*, *BRCA2*, *CSMD3*, *NF1*, *CDK12*, and *RB1* in our cell lines as these genes were the most frequently found somatically mutated in HGS EOC samples as reported by the TCGA [12]. This analysis not only verified the presence of somatic *TP53* mutations and germline *BRCA1*/*BRCA2* mutations identified by other means, but also identified mutations in *CSMD3*, *CDK12* and *RB1* (Table 2). The splice variants in *RB1* and *CDK12* are predicted to affect the production of the encoded protein. The missense p.S646T mutation in *CSMD3*, has not been reported in the Catalogue of Somatic Mutations in Cancer Database (cancer.sanger.ac.uk/cancergenome/projects/cosmic/). Although this amino acid alteration is predicted to be tolerated by SIFT (http://sift.jcvi.org), it is predicted to be probably damaging by PolyPhen-2 (genetics.bwh.harvard.edu/pph2/), based on computational tools that assess the functional effects of amino acid substitutions. The amino acid substitution occurs in a highly conserved region of the gene based on Genomic Evolutionary Rate Profiling (GERP) for mammalian alignments (genome.ucsc.edu). Finally, we also reviewed *PIK3CA*, *CTNNB1*, and *ARID1A*, as well as *KRAS* and *BRAF*, for somatic mutations in these genes have been reported to occur in EOC subtypes and corresponding cell lines most often representing other non-HGS subtype cancers [23]. None of our EOC cell lines harbored mutations in these genes (Table 2).

### Expression of cytokeratin markers, WT-1, p53, PAX8 and HER2 in tumor tissue and cell lines characteristic of HGS EOC

**Figure 4:**
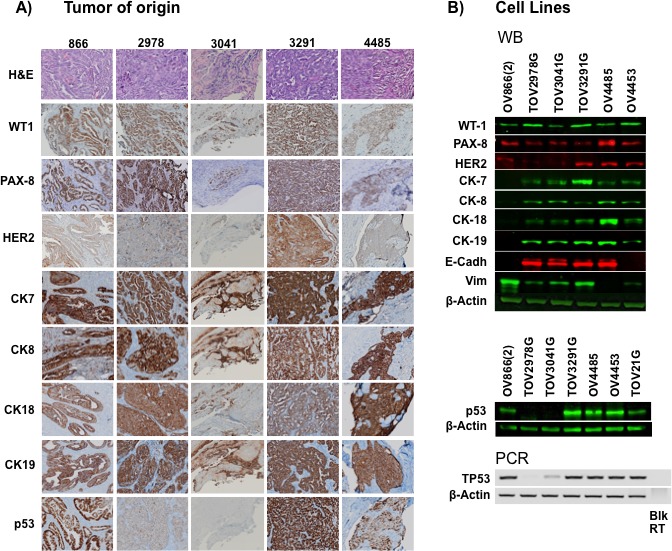
Immunostaining of cell lines and corresponding solid tumors A) Immunohistochemical analysis of formalin-fixed paraffin-embedded solid tumors from patients 866, 2978, 3041, 3291, and 4485 with cytokeratin (CK7, CK8, CK18 and CK19) markers, WT1, PAX8, p53 and HER2. Brown color indicates positive staining, nuclei are counterstained with hematoxylin, and images are at 400x magnification. Topmost images are hematoxylin-eosin images of the corresponding tumor samples. B) Detection of CKs, WT1, PAX8, p53, HER2, E-cadherin and vimentin by Western blot of lysates of cell lines. Lower panel shows an agarose gel of *TP53* PCR products from reversed-transcribed cell line mRNAs. β–actin was used as control in both procedures. Blk = Blank, RT = Reverse transcription

In order to investigate the epithelial origin of the tumors and corresponding cell lines, cytokeratin expression was investigated by immunohistochemistry (IHC) and Western blot (WB) analyses, respectively. IHC was not performed on tissue samples from patient 4453 since no tumor cells were found in a review of the histology. In this case, surgery was performed during the chemotherapy treatment cycles. It is possible that the tumor mass had regressed having already responded to therapy by the time of surgery. HGS diagnosis of this patient was determined from a pre-chemotherapy omentum biopsy. For the available tumor samples, HGS histopathology was confirmed by hematoxylin-eosin staining and all of the cytokeratins investigated (CK7, CK8, CK18 and CK19) were present in the tissue sections (Figure 4A). Cytokeratin expression was observed in protein extracted from five of six EOC cell lines (Figure 4B). Although the 866 tumor tissue shows positive staining for all the cytokeratins, the corresponding OV866(2) cell line exhibited low or barely detectable levels of cytokeratin expression (Figure 4B). This cell line was established from the ascites of this patient collected at recurrence eight months after the primary tumor was collected. E-cadherin, another epithelial marker, was also not detectable in the cell line (Figure 4B). In contrast, high levels of vimentin, a mesenchymal marker, were observed in the protein extracts of this cell line. All the other cell lines in this study expressed either cytokeratins or E-cadherin or both (Figure 4B).

To further characterize the presently described cell lines, expression of WT1 and PAX8, which are markers specific for the classification of the HGS EOC subtype [36-39], was investigated. All tumors and cell lines exhibited positive staining for these proteins and this was observed at distinct intensity levels for each cell line as observed on WB analyses (Figure 4A-B).

Protein expression of the human epidermal growth factor receptor-2 gene, HER2, which has been implicated in malignant transformation, was also used to characterize the cell lines, as overexpression has been reported in 10-20% of serous EOC tumors [40, 41]. HER2 staining by IHC was positive in two solid tumors (866, and 3291) (Figure 4A). HER2 expression was detected in protein extracts of four cell lines (Figure 4B), but intensity of the protein bands was not suggestive of overexpression.

Expression of p53 protein is often indicative of mutation type as was observed in our IHC analysis of *TP53* mutated HGS samples, where missense alleles expressed stable mutated protein in contrast to other mutation types which resulted in no detectable protein (“null” alleles) [35]. Protein expression was detected by WB analysis in all three of the cell lines (OV866(2), TOV3291G, and OV4485) that had a missense mutation (Figure 4B). Interestingly, differential p53 expression was observed in cell lines harboring two distinct intronic *TP53* mutations (splice) of unknown function (based on *TP53* IARC Mutation database). While, p53 mRNA and protein are expressed in the OV4453 cell line (*TP53*:c.376-1 G>A mutation), no mRNA or protein was detectable in lysates from the TOV2978G cell line (*TP53*:c.920-2 A>G mutation) (Table 2 and Figure 4B). Our results showed that despite the absence of p53 protein, the TOV3041G cell line does not harbor a *TP53* mutation by Sanger or whole exome sequencing analyses (Table 2 and Figure 4B). Our RT-PCR results showed a faint band of *TP53* mRNA in this TOV3041G cell line (Figure 4B), which suggests that the absence of p53 was not due to protein degradation but rather mRNA instability or gene silencing. This mRNA instability was not observed in the non-HGS *TP53* wild-type cell line TOV21G [42] that also has detectable p53 protein (Figure 4B), suggesting that this is a feature specific to HGS EOC. IHC results on p53 expression of tumor samples (Figure 4A) were concordant with those of WB in the HGS EOC cell lines.

### Cell lines exhibit aberrant p53 function

**Figure 5:**
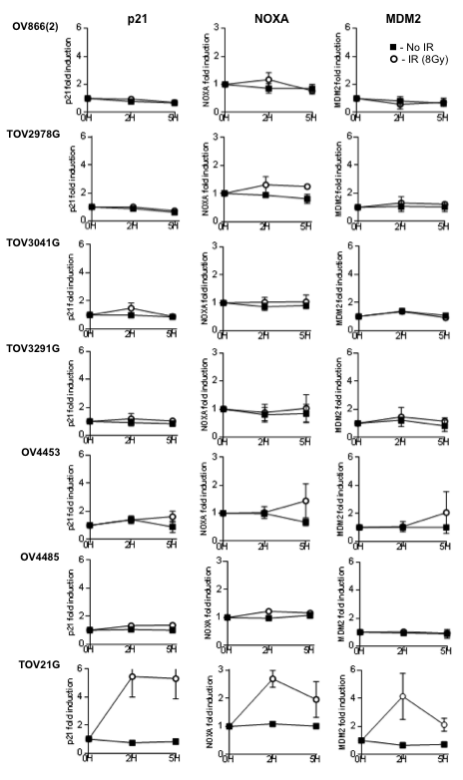
Analysis of p53 function in the different cell lines by RT Q-PCR Gene expression of p53 target genes (p21, NOXA and MDM2) were quantified by RT Q-PCR (see Methods for details) at times 0h, 2h and 5h after 8Gy gamma-irradiation (white circle). Non-irradiated cells were used as controls (black square). Note the increased expression of p53 target genes after DNA damage induced by γ-irradiation in the control p53 proficient cell line TOV21G, and the absence of response for all the HGS EOC cell lines.

The p53 tumor suppressor is a transcription factor that in response to various types of genotoxic stresses transactivates a number of genes by binding to specific DNA sequences [43], thereby arresting cell cycle, repairing damaged DNA, or inducing apoptosis [44]. Among known direct gene targets that are induced by p53 as consequence of genotoxic stress are *CDKN1A*, *PMAIP1* and *MDM2*, which encode the cyclin-dependent kinase inhibitor 1A (p21), phorbol-12-myristate-13-acetate-induced protein 1 (NOXA) and, E3 ubiquitin protein ligase MDM2 proto-oncogene, respectively [45, 46]. Therefore, to further characterize p53 function in our cell lines, we investigated the expression of these genes after inducing DNA damage by 8Gy gamma-irradiation. Figure 5 shows that expression of the p53 target genes analyzed by Q-PCR were not significantly increased after gamma-irradiation in any of the cell lines, although a tendency could be observed for NOXA and MDM2 induction in the OV4453 cell line at a later time point (5h). Nevertheless, all three target genes were efficiently induced in the non-HGS *TP53* wild-type TOV21G cell line even 2h after gamma-irradiation (Figure 5), which expresses the *TP53* mRNA and protein (Figure 4B).

### Cell lines exhibit altered in vitro cell growth phenotypes characteristic of oncogenic potential

The growth characteristics of the new cell lines were compared to other HGS EOC cell lines previously established in our laboratory [11, 27]. The average doubling time ranged from approximately 1.3 to 2.4 days (Table 3), and overlapped the range reported for the other previous HGS cell lines (from 1.5 to 3 days) [11, 27]. Saturation densities were similar for four of the cell lines (OV866(2), TOV2978G, TOV3291G, OV4453) and this was similar to previously described saturation densities (1.5-3.5 × 10^6^ cells) of other HGS EOC cell lines [11, 27]. However, the saturation densities of the TOV3041G and OV4485 cell lines were significantly higher when compared to any of the other cell lines (Table 3).

**Table 3:** The *in vitro* and *in vivo* growth characteristics of the cell lines

Cell line		OV866(2)	TOV2978G	TOV3041G	TOV3291G	OV4453	OV4485
Growth characteristics in cell culture	Doubling time (days) AVG±SEM	1.77±0.32	1.87±0.11	1.33±0.02	2.28±0.19	2.44±0.03	1.85±0.29
	Saturation density (1×10^6^ cells) AVG±SEM	3.31±0.02	2.04±0.12	8.10±0.87*	2.16±0.04	3.18±0.12	5.95±0.64*
	Number of passages to date	>P200	P100	P100	P100	P100	P90
Spheroid formation		semi compact	aggregate	compact	semi compact	aggregate	semi compact
Scratch assay migration velocity (μm/h) AVG±SEM		61.8±10.7*	17.2±0.75	15.2±2.9	21.7±2.7	17.4±1.3	3.9±0.6*
Growth efficiency in soft agar (colony count) AVG±SEM		34.4±7.3	N/D	N/D	N/D	21.1±5.0	N/D
Carboplatin IC_50_ (μM) AVG±SEM		32.1±7.1	0.76±0.24	0.81±0.64	2.7±0.9	0.23±0.07	6.1±0.27
Number of mice (n=5) with tumor xenograft	Subcutaneous injection site	2	0	0	0	5	5
	Intraperitoneal injection site	4	-	-	-	5	5
Mean time of tumor appearance (days)	Subcutaneous injection site	250	N/A	N/A	N/A	138	98
	Intraperitoneal injection site	150	-	-	-	70	50

N/D = non detectable, N/A = not applicable, - = experiement not conducted

* denotes statistical differences (p<0.05, Student's t-test) when compared to each of all the other cell lines

**Figure 6:**
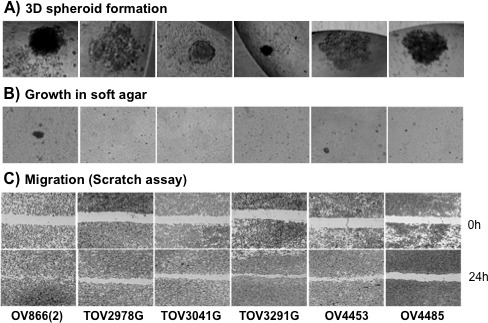
*In vitro* characterization of cell lines using diverse oncogenic assays A) Spheroid formation of cell lines after 5-7 days in OSE culture media using the inverted droplet technique. Photos are representative of observations from three independent experiments. B) Anchorage-independent growth in soft agar. Pictures show representative images of colonies formed in the agar after two-weeks of culture from three independent experiments performed in triplicate. C) Migration evaluation by the wound-healing scratch assay. Photos show representative images of monolayer cell cultures at 0h and 24h after the scratch was performed. Three independent experiments were performed in triplicate. In all experiments, pictures of different cell lines were taken at the same magnification.

The ability of each cell line to form spheroids was measured using the hanging droplet method as previously described [47]. Observed spheroid characteristics include overall shape, compaction, cell aggregates and consistency across multiple experiments, as conducted with other EOC cell lines derived in our laboratory [11, 27, 47]. Although there were some variations among the replicates, only the TOV3041G cell line consistently formed compact spheroids. In contrast, the OV866(2), TOV3291G and OV4485 cell lines formed loosely compact spheroids with irregular margins and the TOV2978G and OV4453 cell lines formed numerous individual small aggregates (Figure 6A, Table 3).

We next measured the ability of the cell lines to grow in an anchorage independent environment by culturing the cells in soft agar. Only the OV866(2) and OV4453 cell lines were able to clearly form colonies in soft agar (Figure 6B, Table 3). The sizes of the colonies were smaller than the previously described HGS TOV1946 cell line but similar to that of the TOV2223G [11]. The number of colonies formed by the OV866(2) and OV4453 cell lines were in the same range as that of other HGS cell lines derived in our laboratory [11, 27].

The migration potential of the cell lines was measured using an established scratch migration assay [11, 27, 48]. Migration velocity was calculated based on the time it took for the cells to cover the gap created by removing the cells after confluence had been reached. Four of the cell lines, TOV2978G, TOV3041G, TOV3291G and OV4453, had similar migration velocity, exhibiting a range of 15-21 μm/h, and almost filling the gap within 24h. In contrast, the OV866(2) cell line had a significantly higher migration speed (62 μm/h) when compared to any of the other cell lines, completely covering the gap in 24h; and the OV4485 cell line had a significantly lower migration speed (Figure 6C, Table 3).

### Carboplatin sensitivity of the cell lines

The carboplatin response of the cell lines was measured by a clonogenic assay. Our results indicate that the OV866(2) cell line was strongly resistant to carboplatin, exhibiting the highest IC_50_ (32 μM) among all the tested cell lines (Table 3). This finding is consistent with the characteristic of the patient sample from which it was derived, i.e. a second ascites from a recurrent resistant disease. Two other cell lines showed medium resistance to carboplatin (TOV3191G and OV4485) and three were very sensitive (TOV2978G, TOV3041G and OV4453) (Table 3). Interestingly, the BRCA1 mutated cell line (OV4485) shows resistance to carboplatin (IC_50_ = 6 μM), making it a good model to study new therapeutic strategies for platinum resistant BRCA mutated HGS EOC cases.

### BRCA1 and BRCA2 mutated cell lines are tumorigenic in mouse xenograft models

**Figure 7:**
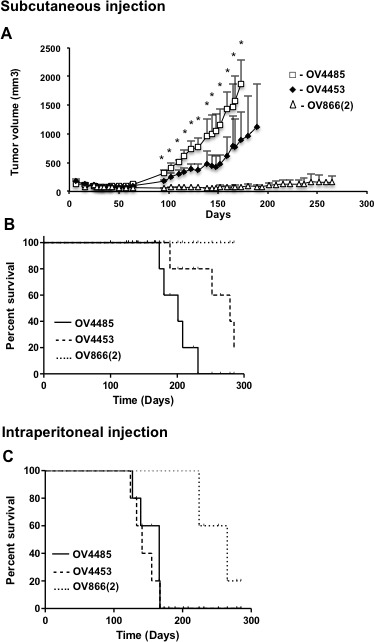
*In vivo* SCID mouse xenograft tumor formation A) Tumor volume of OV4485 (white square), OV4453 (black lozenge) and OV866(2) (white triangle) cell lines injected subcutaneously in SCID mice (n=5). Points represent average ± SEM and curves were plotted up until the first animal was sacrificed when end-point limits were attained. * denotes statistical differences (p<0.05, Student's t-test). B and C) Kaplan-Meyer survival curves of mice injected subcutaneously (n=5) or intraperitoneally (n=5) in SCID mice with OV4485 (solid line), OV4453 (dashed line) and OV866(2) (assured line) cell lines. Survival was followed over a period of approximately 10 months and each event was considered as the time of sacrifice at end-point limits.

The *in vivo* growth potential of all the cell lines was determined by subcutaneous injection into SCID mice (n = 5 mice for each cell line). Only the HGS cell lines OV4453 and OV4485, which were derived from *BRCA2* and *BRCA1* mutated cases, respectively, formed tumors at subcutaneous injection sites in all the SCID mice (5/5 mice) (Figure 7A-B, Table 3). Tumor growth of the OV4485 cell line was significantly faster and mice had a poorer overall survival than that observed with the OV4453 cell line tumorigenicity assays. Tumors started to appear (volume ≥ 300 mm^3^) as early as 98 and 138 days for the OV4485 and OV4453 cell lines, respectively, and reached endpoint limits allowable for tumor growth (2500 mm^3^) at 170-230 and 190-320 days, respectively. Figure 7A also shows the tumor growth of the OV866(2), which was the only other cell line to produce tumors. However, the tumor mass at subcutaneous injection sites reached a maximum of 390 mm^3^ in 265 days, and this was observed only in two of five mice (Figure 7A-B, Table 3).

We also investigated the capacity of the tumorigenic cell lines, OV866(2), OV4453 and OV4485, to form tumors and produce ascites at intraperitoneal injection sites in SCID mice. The experimental endpoints for these experiments were within ethical limits and included palpable peritoneal tumors, bloated abdomen and stress behavior. The survival curves are shown in Figure 7C. For this model, the OV4453 and OV4485 cell lines induced tumors and produced ascites in all mice at a similar rate, and this was faster than that observed with the OV866(2) cell line, which induced intraperitoneal tumors and ascites in four of five mice (Table 3).

## DISCUSSION

The genomic instability and aberrant karyotype of HGS EOC tumors has been well described and is distinct from that observed in the other EOC histotypes, which often exhibit a more stable genome [49, 50]. The recent study from the TCGA also highlighted the high molecular genetic heterogeneity among HGS tumors, although specific genomic anomalies recurred at variable frequencies [12]. Their analyses delineated four transcriptional subtypes, three microRNA subtypes, four promoter methylation subtypes and a transcriptional signature associated with survival in an analysis of over 300 HGS cases. There were recognizable features such as high somatic copy number alterations, an overall high frequency (96%) of somatic *TP53* mutations, *BRCA1*/*BRCA2* germline/somatic mutations or silencing of gene expression in approximately 33% of the tumors, and somatic mutations in *CSDM3*, *NF1*, *CDK12*, and *RB1* that each occurred at low frequency [12]. Recently, the genetic and genomic profiles of several EOC cell lines were compared to the TCGA data in order to gather evidence to verify the suitability of these cells as HGS EOC models [23]. As criteria the authors used the presence of the above-mentioned common features of HGS tumors as well as the absence of mutations in seven specific genes (*PIK3CA*, *PTEN*, *KRAS*, *BRAF*, *ERBB2*, *CTNNB1* and *ARID1A*) that have been reported to occur more often in other EOC subtypes. Few cell lines examined in their study exhibited the molecular genetic features characteristic of HGS EOC samples. In contrast, we have shown that all of our six newly derived cell lines exhibit genetic and genomic features characteristic of HGS samples, suggesting that they have a high probability of being of HGS EOC tumor origin. Namely, that they have high copy number alterations and/or harbor *TP53* mutations. Some of the cell lines were derived from patients with germline *BRCA1*/*BRCA2* mutations. Moreover, some of these cell lines had somatic mutations in *CSDM3*, *RB1*, and *CDK12*, genes that have been shown to exhibit a low but recurrent mutation frequency in HGS EOC tumors. Although we have shown that the molecular genetic spectrum of each of our EOC cell lines is unique, collectively they reflect the heterogeneity of HGS EOC disease. These novel HGS EOC cell lines provide novel research tools for investigating the most common and lethal form of EOC.

The *BRCA1*/*BRCA2* mutation harboring cell lines (OV4485 and OV4453) also each have a somatic *TP53* mutation, and they were the only cell lines that exhibited robust tumorigenic potential in SCID mice. These genetic features makes them attractive models to study novel therapeutics targeting HGS EOC patients harboring *BRCA1*/*BRCA2* mutations, such as the inhibitors of poly (ADP-ribose) polymerase, a class of drugs currently in phase II/III clinical trials in EOC [51-54] that includes Olaparib which has been recently approved by the US Food and Drug Administration as maintenance therapy in *BRCA*-mutated platinum-sensitive HGS EOC [55]. In the present study we also described a new *BRCA1* mutation located at the intron/exon boundary of exon 15 (IVS14-1 G>T). The establishment of the OV4485 cell line made it easier to characterize this mutation and we showed that it induced alternative splicing as well as instability of the *BRCA1* mRNA (Figure 2). The frequency of this mutation is unknown, however no other mutation carriers were reported in a *BRCA1*/*BRCA2* mutation screen of 136 breast and/or ovarian cancer families of French Canadian descendent [56, 57]. Further studies are warranted to investigate the clinical relevance of this new *BRCA1* mutation. In contrast, the nonsense *BRCA2*:G6085T (E1953X) found in the OV4453 patient and corresponding cell line is a pathogenic germline mutation that has been found to recur in the French Canadian population [28, 56]. Until recently, only five HGS EOC cell lines harboring *BRCA1*/*BRCA2* mutations from EOC samples had been described in the literature [23, 25, 26]. During the course of preparing this manuscript, two other *BRCA* mutated HGS EOC cell lines were reported [58]. Nevertheless, none of these cell lines harbor the mutations found in our study. Based on published reports, only the PEO1 (a *BRCA2* mutated) cell line is tumorigenic in xenografted mice [59]. Here we show that both of our *BRCA1* and *BRCA2* mutated cell lines (OV4485 and OV4453 respectively) form tumors in mice. To our knowledge, the OV4485 cell line is the only *BRCA1* mutated HGS EOC cell line with *in vivo* tumorigenic properties in SCID mice. This cell line also showed resistance to carboplatin (Table 3), making it a very suitable *in vivo* model to study new therapies for platinum resistant BRCA mutated cases.

The OV866(2) cell line was the only line expressing markers of epithelial-mesenchyme-transition (EMT), as reflected by the absence of cytokeratins (CK7, CK8, CK18, CK19) and E-cadherin expression, and strong vimentin expression (Figure 4). This cell line also showed the highest carboplatin IC_50_, anchorage-independent growth and cell invasion when compared to the other five cell lines (Figure 6 and Table 3). Research has shown that opposing levels of E-Cadherin and vimentin occur in cells that have undergone EMT [60, 61]. Therefore, this new cell line may be an interesting model to study EOC processes related to peritoneal metastasis and invasion, because there is evidence that EMT transition might be involved in this tumor spread [62]. All the other five cell lines showed patterns of epithelial origin as suggested by the presence of cytokeratins and/or E-cadherin expression.

The TOV3041G cell line was derived from a rare case of HGS EOC where a somatic *TP53* mutation was not detected by our mutation screening. However, we have demonstrated the absence of both p53 mRNA and protein as well as the impaired p53 function in this cell line. Therefore, although *TP53* mutation analysis suggests that it harbors a wild-type allele, this cell line has features characteristic of a p53-null mutation. The p53 protein functions as a transcriptional factor with a crucial role in cellular stress response (reviewed in [63-65]). Owing to its critical function in controlling cell survival or death, strict regulation of p53 levels and activity is crucial. At the protein level, p53 is kept under tight control by multiple mechanisms, such as the regulation by MDM2, which ubiquitinates p53 and targets it for proteasome-mediated degradation (reviewed in [66, 67]). Furthermore, mRNA stability and *TP53* transcription is also subject to regulation by a number of factors, such as BCL6, PAX2, PAX8, Wrap53, and specific miRNAs, all of which are reported to repress *TP53* mRNA expression or to render its mRNA unstable [68-70]. Our results suggest that in the TOV3041G cell line, the *TP53* regulation occurs at the mRNA level rather than the protein level, requiring further investigation for the underlying mechanism of inactivation. In the case of the TOV2978G cell line, the intronic *TP53* mutation seems to also affect transcription, since neither mRNA nor protein were detected in this cell line. Hence, our newly described cell lines also reflect the multi facets of the HGS EOC disease in which *TP53* gene is either mutated or silenced [12, 32, 71].

Interestingly, the two p53-deficient cell lines (TOV2978G and TOV3041G) also did not express BRCA1 mRNA or protein (Figure 2), despite the fact that they do not harbor *BRCA1* mutations. These cell lines would then represent HGS EOC with silenced *BRCA* gene (possibly by epigenetic mechanisms), which is another very common feature of this disease [12]. Identifying two cell lines with epigenetically silenced *BRCA1* is expected given the apparently high frequency of *BRCA1* promoter hypermethylation, estimated as 12% of HGS EOC tumors observed by the TCGA study [12]. Finally, the TOV3291G harbors a *TP53* mutation, a complex CNA pattern and highest ploidy of the six cell lines, and is the only one to have a *CDK12* mutation. Moreover, we have demonstrated that all of our cell lines do not have a functional p53, a feature characteristic of HGS EOCs.

The presently described EOC cell lines did not exhibit molecular genetic events characteristic of other histological subtypes of EOC. None of them harbored somatic activating mutations in *KRAS* or *BRAF* (Table 2), a feature found in LGS tumors and other subtypes of EOC [12, 18, 72]. They also did not harbor mutations in *ARID1A*, *CTNNB1*, or *PIK3CA* that has been observed in other subtypes of EOC, particularly endometrioid adenocarcinomas [23]. HER2 overexpression was not observed in these cell lines, which has been shown to be a feature of clear cell EOC [40, 41, 73, 74]. Therefore, our models can be efficiently distinguished from other EOC sub-types, further highlighting the importance of these cell lines as truly representative HGS EOC models.

## MATERIAL AND METHODS

### Patient and sample data

Tumor and ascites samples were collected from patients following informed consent from the Centre hospitalier de l'Université de Montréal (CHUM), Division of Gynecologic Oncology. The study was approved by the Comité d'éthique de la recherche du CHUM, the institutional ethics committee. Stage was determined at time of surgery by a gynecologic oncologist. Histology and tumor grade were determined by a gynecologic-oncology pathologist using criteria consistent with the International Federation of Gynecology and Obstetrics (FIGO) classification [75].

### Cell line establishment and culture conditions

In total, six cell lines were derived from samples from six patients, 866, 2978, 3041, 3291, 4453 and 4485. All cell lines were maintained in a low oxygen condition of 7% O_2_, and 5% CO_2_ and grown in complete OSE medium, which includes OSE medium (Wisent, St-Bruno, QC), 10% FBS, 0.5 μg/mL amphotericin B (Wisent) and 50 μg/mL gentamicin (Gibco®, Life Technologies Inc., Burlington, ON). The solid ovarian tumor (TOV)-derived cell lines (TOV2978G, TOV3041G, TOV3291G) were established using the scrape method as previously described [11, 76]. Briefly, tumor tissue was scraped into a 100 mm plate with complete OSE medium and maintained for 40 days with the medium replaced weekly. Cells were passaged at near confluence, and were considered immortal when passaged over 50 times. The OV cell lines (OV866(2), OV4453, OV4485) were established from the cellular fraction of ascites collected by centrifugation [11, 76]. The cell lines derived from ascites cells were maintained as above for the TOV derived cell lines. Although to date each cell line has reached at least 90-100 passages, most assays were conducted on cell lines between passage 60 and 80.

### Cell growth rates

Growth rates were assessed as previously described [11, 42]. Briefly, cells were seeded on day 0 in 100 mm^3^ dishes (2 × 10^5^ cells per dish). The complete OSE media was replaced every three days for the duration of the experiment. Three times a week from day one to 14, cells were trypsinized, resuspended in media and counted using a hemocytometer. Saturation density was defined as the mean maximum number of cells counted at confluence. Each experiment was performed in triplicate, and repeated twice. Doubling times were determined using a publically available algorithm (www.doubling-time.com).

### Antibodies

WB and IHC analyses were performed using the following antibodies: p53 (D0-1, sc-126, Santa Cruz Biotechnology, Dallas, TX); HER2/ErbB2/Neu (C-18, sc-284, Santa Cruz Biotechnology, for WB; and OP15, Calbiochem®, Millipore (Canada) Ltd., Etobicoke, ON, for IHC); Cytokeratin (CK) 7 (Ab-2, MS-1352-P, Thermo Scientific, Waltham, MA); CK8 (Ab-4, MS-997-P, Thermo Scientific); CK18 (DC-10, sc-6259, Santa Cruz Biotechnology); CK19 (Ab-1, MS198-P, Thermo Scientific), WT1 (6F-H2, 05-753, Millipore (Canada) Ltd.), BRCA1 (OP92, MS110, Calbiochem®, Millipore (Canada) Ltd.), Vimentin (V9, sc-6260, Santa Cruz Biotechnology), E-cadherin (24E10, #3195, Cell Signaling Technology Inc., Danvers, MA), PAX8 (10336-1-AP, Proteintech, Chicago, IL) and beta-actin (AC-15, ab6276, Abcam Inc., Toronto, ON, Canada).

### Western blot analysis

In general (with the exception of BRCA1 detection, see below), 30 micrograms of total protein extracts were electrophoresed in 10% SDS-polyacrylamide gels. Proteins were then transferred onto nitrocellulose membranes using the Trans-Blot Turbo Transfer System (BioRad, Mississauga, ON) and membranes were blocked with a specific Odyssey® blocking buffer (LIC-927-40010, Mandel Scientific, Guelph, ON) for immunofluorescence detection. Membranes were then probed with primary antibodies in 1% BSA-Phosphate buffered saline (PBS)-Tween at the following dilutions: 1/10000 for beta-actin, 1/1000 for CK8, 1/2000 for CK7 and CK18, 1/500 for p53, E-cadherin, and vimentin, 1/200 for HER2, 1/5000 for PAX8 and 1/250 for WT1. Primary antibody labeling was detected with an IR-Dye-fluorochrome conjugated secondary antibody and visualized with the LI-COR Odyssey apparatus (Mandel Scientific). Antibody against beta-actin was used as a loading control.

For BRCA1, 60 micrograms of total protein extracts were electrophoresed in 4%-15% pre-cast gels (Bio-Rad). Proteins were then transferred onto nitrocellulose membranes at 400mA (overnight, 4°C) using the conventional blotting system. Membranes were then blocked with 5% milk PBS-Tween and probed with primary antibody diluted 1/1000 in 1% BSA PBS-Tween (overnight, RT). Antibody labeling was detected with a HRP-conjugated secondary antibody and visualized by the enhanced chemiluminescence (ECL) method.

### Immunohistochemistry

Tissue sections (4μm) were stained with the Benchmark XT automated stainer (Ventana Medical System Inc., Tucson, AZ). Antigen retrieval was obtained using Cell Conditioning #1 or #2 (Ventana Medical System Inc.) depending on the protein target (see Supplementary Table S1). Pre-diluted antibodies were manually added to the slides and incubated at 37°C (see Supplementary Table S1 for details). Reactions were performed using the UltraView universal DAB detection kit (Ventana Medical System Inc.). Counterstaining was achieved with hematoxylin and bluing reagent (Ventana Medical System Inc.). All sections were scanned using a VS-110 microscope (Olympus, Center Valley, PA) with a 20X 0.75NA objective and resolution of 0.3225 μm, allowing images to be viewed at a 400x magnification. The OlyVIA software (Olympus) was used for image analysis.

### Targeted mutation analysis of TP53, KRAS, BRAF, BRCA1 and BRCA2

Mutation analyses were performed using DNA that was extracted from the cell lines (passages 52 to 108) as described previously [76]. Mutation analyses were designed to detect variants in the protein coding exons 2 to 11, and adjacent splice sites of *TP53*, and the common mutations occurring in either exon 2 of *KRAS* or exons 11 and 15 of *BRAF*. Mutation analysis was performed using PCR-based assays followed by bidirectional sequencing using the 3730XL DNA Analyzer system (Applied Biosystems®, Life Technologies Inc., Burlington, ON) at the McGill University and Genome Quebec Innovation Center (Montreal, QC, Canada) as previously described [18]. Sequence chromatograms were compared with NCBI reference sequence (RefSeq) reported in GenBank: NM_000546.4 (*TP53*), NM_004985.3 (*KRAS*) and NM_004333.4 (*BRAF*), and the genomic structures available from the February 2009 GRCh37/hg19 assembly of the human reference genome. Sequence variants were compared with those reported in the SNP Database (www.ncbi.nlm.nih.gov/SNP). In addition, *TP53* variants were evaluated based on information in the International Agency for Research on Cancer (IARC) TP53 Database (www-p53.iarc.fr).

The ovarian cancer specimens were obtained from French Canadian women, a population known to have specific recurrent *BRCA1* and *BRCA2* mutations due to common ancestors [29]. DNA from peripheral blood lymphocytes was investigated for the most common mutations in *BRCA1* (C4446T and 2953delGTAinsC) and *BRCA2* (8765delAG, G6085T and 3398delAAAAG) using established PCR-based screening assays [29]. Due to limited availability of peripheral lymphocyte DNA from patient 866, the OV866(2) cell line DNA was also investigated. DNA from peripheral blood lymphocytes of the remaining five patients were also investigated by the Luminex platform, which screens for a broader panel of 19 *BRCA1*/*BRCA2* mutations (including the above recurrent mutations) that represent the majority of pathogenic mutations identified in the French Canadians of Quebec as described [29, 56, 77]. Through the Hereditary Cancer Clinic affiliated with CHUM, DNA from peripheral blood lymphocytes from patients 4453 and 4485 were also genetically tested by BRAC*Analysis*^®^, a comprehensive mutation analysis for mutations in *BRCA1* and *BRCA2* (www.myriad.com). All variants were verified by bidirectional Sanger sequencing as described above, and compared with *BRCA1* U14680 or *BRCA2* U43746 GenBank sequences (www.ncbi.nlm.nih.gov), and the GRCh37/hg19 assembly of the human reference genome. The Human Genome Variation Society (HGVS) (www.hgvs.org/mutnomen/) designation is provided for all mutations investigated and identified in this study. However, for historical reasons, the original *BRCA1*/*BRCA2* mutation nomenclature is referred to throughout in cases where a recurrent mutation was identified. The variants were also investigated in the patient-matched cell line.

### Whole exome sequencing analyses

Libraries for whole exome sequencing were prepared from 500 ng of DNA from the six cell lines using the NimbleGen SeqCap EZ Human Exome Library v3.0 kit (Roche NimbleGen, Inc., Madison, WI), followed by paired-end, 100bp sequencing on the HiSeq 2000 (Illumina, Inc., San Diego, CA) at an equivalent of three samples per lane, according to recommended protocols at the McGill University and Genome Quebec Innovation Centre. Alignment to the human genome (NCBI37/hg19) and variant calling was performed using Trimmomatic (to remove adaptors and trimming reads to minimum phred score of 30) (www.usadellab.org/cms/?page=trimmomatic), BWA (alignment) (bio-bwa.sourceforge.net/), GATK (realignment of INDELs, base quality recalibration) (www.broadinstitute.org/gatk/) and Picard (mate-pair recalculation, mark duplicates) (broadinstitute.github.io/picard/) software programs. Variant calling was performed using Samtools and Bcftools (www.htslib.org/), and annotated using SnpEff (snpeff.sourceforge.net/) and dbNSFP (sites.google.com/site/jpopgen/dbNSFP). Additional variant filtering included: minimum read depth of 10 reads, minimum allele frequency of 20%, and a minor allele frequency (MAF) of less than 2% in European Americans from the Exome Variant Server (evs.gs.washington.edu/EVS/). The sequencing reads for the variants in candidate genes were also reviewed manually using the Integrative Genomics Viewer (IGV) (www.broadinstitute.org/igv).

### TP53 and BRCA1 cDNA polymerase chain reaction

Total RNA was extracted from all six cell lines by the TRIzol Reagent (Invitrogen™, Life Technologies Inc., Burlington, ON) followed by the RNeasy kit (Qiagen Inc., Toronto, ON, Canada). One microgram of total RNA was subjected to reverse transcription using the QuantiTect Reverse Transcription Kit (Qiagen Inc.). One microliter of the reverse-transcribed product either diluted (1/10, for *TP53* or beta-actin amplification) or not (for *BRCA1* amplification) was subjected to standard polymerase chain reaction (PCR) using the Phusion High-Fidelity PCR kit (New England BioLabs, Whitby, ON), according to the manufacturer's instructions. Two pairs of primers were used for *BRCA1* amplification, one covering exons 13 to 15: forward, 5′-Gactcttctgcccttgagga-3′ and reverse, 5′-ctgttgctcctccacatcaa-3′; and another covering exons 14-16: forward, 5′-Ggcctttctgctgacaagtt-3′ and reverse, 5′-aattctggcttctccctgct-3′. Primers for *TP53* were: forward, 5′-GGAAGACTCCAGTGGTAATCTA-3′ and reverse, 5′-TTGGGCAGTGCTCGCTTA-3′, which amplify all *TP53* variants; and for the beta-actin control gene: forward, 5′-ACTCTTCCAGCCTTCCTTCC-3′ and reverse, 5′-GTACTTGCGCTCAGGAGGAG-3′. PCR products of *BRCA1* amplifications were subjected to bidirectional Sanger sequencing (Centre de recherche du Centre hospitalier de l'Université de Laval), and sequence chromatograms were compared with *BRCA1* U14680 GenBank sequence (www.ncbi.nlm.nih.gov), and the GRCh37/hg19 assembly of the human reference genome.

### Genomic analyses using SNP arrays

Genome-wide chromosomal anomalies, such as chromosomal breaks, CNA and LOH, were inferred in the six cell lines using the HumanOmni2.5Exome BeadChip (Illumina, Inc.). This BeadChip assays 2,567,845 markers, with an average spacing of 1.14 Kb per marker (median spacing is 0.57 Kb). Genotyping, using 200 ng of DNA, and scanning were performed at the McGill University and Genome Quebec Innovation Centre. All samples had call rates (the percentage of valid genotype calls) within the range of 0.945 and 0.993 (average 0.968). Genotyping analysis was performed using the Genome Viewer module in GenomeStudio software (Illumina, Inc.). The software aligns genotyping data for each marker with genomic map coordinates based on the NCBI37/hg19 assembly of the human reference sequence (genome.ucsc.edu/cgi-bin/hgGateway). An image file was created for inferring genomic rearrangements based on the allele frequency (BAF) and copy number (LogR ratio) for each marker assayed. Homozygous deletions were inferred based on Log R ratios less than −2 for at least five adjacently mapped markers. Normalized SNP intensity files were also analyzed by the allele-specific copy number analysis of tumors (ASCAT) algorithm (heim.ifi.uio.no/bioinf/Projects/ASCAT/, and bcb.dfci.harvard.edu/~aedin/courses/Bioconductor/rcourse.r), modified to allow for 100% aberrant cell fraction [78]. ASCAT first segments the genome, and then interprets the abnormalities in each segment to establish the copy number of each parental chromosome, based on the ploidy and aberrant cell fraction. The CNA and LOH status of each segment were visualized with IGV. Gain and loss for loci of interest were inferred relative to ploidy.

### Assessment of p53 function by real time quantitative polymerase chain reaction (Q-PCR)

Cells were seeded on 35 mm^3^ plates, grown until 80% confluence, gamma-irradiated at 8Gy, and then harvested at 0h, 2h and 5h after irradiation. Total RNA was extracted using TRIzol Reagent (Invitrogen™, Life Technologies Inc.) followed by the RNeasy kit (Qiagen Inc.). One microgram of total RNA was subjected to reverse transcription using the QuantiTect Reverse Transcription Kit (Qiagen Inc.). One microliter of the reverse-transcribed product was diluted (1:10) and subjected to Q-PCR using sequence-specific primers (400 nM) and the SYBR Select Master Mix (Applied Biosystems^®^, Life Technologies Inc.). Sequence primers for target genes were: p21, forward, 5′-ACCCTAGTTCTACCTCAGGC-3′, reverse, 5′-AAGATCTACTCCCCCATCAT-3′; NOXA, forward, 5′-AGAGCTGGAAGTCGAGTGT-3′, reverse, 5′-GCACCTTCACATTCCTCTC-3′; MDM2, forward, 5′-ATCTTGGCCAGTATATTATG-3′, reverse 5′-GTTCCTGTAGATCATGGTAT-3′; and GAPDH, forward, 5′-GGGAAGGTGAAGGTCGGAGT-3′, reverse, 5′-TTGAGGTCAATGAAGGGGTCA-3′. The Q-PCR was achieved with the Applied BioSystems^®^ Step One Plus apparatus. Thermocycling conditions were one UDG activation cycle at 50°C for 2 min and one AmpliTaq activation plus denaturation cycle at 95°C for 2 min followed by 40 cycles at 95°C for 15sec, 60°C for 1 min and 72°C for 30 sec. Gene expression values were normalized to the GAPDH gene. Two or three independent experiments were performed in duplicate.

### Spheroid assay

A spheroid assay was performed to determine the ability of cell lines to generate three-dimensional structures in the form of aggregates, as previously described [47, 79]. Briefly, 4,000 cells were suspended in 16 μl of complete OSE medium and placed on the cover of non-coated plastic tissue culture plates that were subsequently inverted. PBS was added to the bottom plate to prevent dehydration of droplets. Spheroid formation ability was assessed after 5-7 days of incubation at 37°C, 7% O_2_, 5% CO_2_, with spheroid formation of the cell lines being classified concordantly with previous research [11, 47].

### Anchorage independent growth in soft agar

Cell lines were assayed for their ability to grow in anchorage independent conditions by culturing 2 × 10^4^ cells in a semi-solid media containing noble agar. Cells suspended in 0.33% w/v agar in complete OSE medium were applied over a base layer comprised of 0.66% w/v agar in complete OSE medium [27, 76]. Cells were cultured in soft agar for three weeks, and colonies were stained with 0.1% crystal violet and 2% methanol solution (for 2h, RT), photographed and counted. Three independent experiments were performed in triplicate.

### Wound-healing assay

Migration potential was evaluated using the scratch assay method as previously described [11, 27, 48]. Cells were grown to confluence in 12-well culture plate dishes. Using a 200 μl pipette tip, a wound was produced in the middle of the monolayer. The adherent monolayer was washed with PBS to remove non-adherent cells and complete OSE media was then added. At each time point (0h, 6h, 12h, 24h, 30h, 36h, 48h), cells were stained with 0.5% methylene blue and 50% methanol solution for 5 minutes and images were taken using the Nikon Eclipse TE300 microscope (Nikon Instruments Inc., Melville, NY). The software NPS-Elements BR (Nikon Instruments Inc.) was used to measure the residual scratch width at each time point and migration velocity was calculated as μm/h.

### Clonogenic survival assays

Carboplatin sensitivity of cell lines was assessed using a clonogenic assay [27, 80]. Briefly, cells were seeded in a 6-well dish at a density of cells/well that allowed the formation of individual colonies (TOV3041G, 750 cells/well; OV866(2), TOV2978G and TOV3291G, 1500 cells/well; TOV3041G; OV4453 and OV4485, 2000 cells/well). Cells were seeded and allowed to adhere for 16 hours in a 37°C, 5% CO2, 7% O2 incubator after which the media was removed and replaced with OSE complete media containing carboplatin (0–100 μM) (Hospira Healthcare Corporation, Saint-Laurent, QC). Cells were incubated with the drug for 24 hours. The drug was then removed and OSE complete media was added. When colonies became visible at a 2X magnification plates were fixed with cold methanol and colored with a mix of 50% v/v methanol and 0,5% m/v blue methylene (Sigma–Aldrich Inc., St. Louis, MO). Colonies were counted under a stereomicroscope and reported as percent of control. IC_50_ values were determined using Graph Pad Prism 5 software (GraphPad Software Inc., San Diego, CA). Each individual experiment was performed in triplicate and repeated three times.

### In vivo growth in SCID mice

All animal studies were approved by the Institutional Committee on Animal Protection (CIPA) protocol according to the Canadian Council on Animal Care. The tumorigenic potential of cell lines was assessed based on their ability to form tumors in 6-week old female SCID (severe combined immunodeficiency) mice (Charles River Laboratories, Saint-Constant, QC) at subcutaneous left gluteal injection (sc.) or intraperitoneal sites (ip.). A volume of 200 μl was injected in each mouse and consisted of 5 × 10^6^ cells resuspended in 100 μl of cold D-PBS (Gibco®, Life Technologies Inc.) and 100 μl of either Matrigel (Becton-Dickinson, Franklin Lakes, NJ) for sc. injections or D-PBS for ip. injections. The animals were housed under sterile conditions in a laminar flow environment with *ad libitum* access to food and water. Tumor formation was assessed twice a week for over 200 days. Animals were sacrificed before neoplastic masses reached limit points established by the CIPA according to the Canadian Council on Animal Care.

## SUPPLEMENTARY MATERIAL FIGURE AND TABLE



## References

[1] Siegel RL, Miller KD, Jemal A (2015). Cancer statistics. CA Cancer J Clin.

[2] Gurung A, Hung T, Morin J, Gilks CB (2013). Molecular abnormalities in ovarian carcinoma: clinical, morphological and therapeutic correlates. Histopathology.

[3] Le Page C, Provencher D, Maugard CM, Ouellet V, Mes-Masson AM (2004). Signature of a silent killer: expression profiling in epithelial ovarian cancer. Expert Rev Mol Diagn.

[4] Bookman MA (2012). First-line chemotherapy in epithelial ovarian cancer. Clin Obstet Gynecol.

[5] Stuart GC, Kitchener H, Bacon M, duBois A, Friedlander M, Ledermann J, Marth C, Thigpen T, Trimble E (2011). 2010 Gynecologic Cancer InterGroup (GCIG) consensus statement on clinical trials in ovarian cancer: report from the Fourth Ovarian Cancer Consensus Conference. Int J Gynecol Cancer.

[6] Coleman MP, Forman D, Bryant H, Butler J, Rachet B, Maringe C, Nur U, Tracey E, Coory M, Hatcher J, McGahan CE, Turner D, Marrett L, Gjerstorff ML, Johannesen TB, Adolfsson J (2011). Cancer survival in Australia, Canada, Denmark, Norway, Sweden, and the UK, 1995-2007 (the International Cancer Benchmarking Partnership): an analysis of population-based cancer registry data. Lancet.

[7] Cho KR, Shih Ie M (2009). Ovarian cancer. Annu Rev Pathol.

[8] Prat J (2012). New insights into ovarian cancer pathology. Ann Oncol.

[9] Wei W, Dizon D, Vathipadiekal V, Birrer MJ (2013). Ovarian cancer: genomic analysis. Ann Oncol.

[10] Kuo KT, Guan B, Feng Y, Mao TL, Chen X, Jinawath N, Wang Y, Kurman RJ, Shih Ie M, Wang TL (2009). Analysis of DNA copy number alterations in ovarian serous tumors identifies new molecular genetic changes in low-grade and high-grade carcinomas. Cancer Res.

[11] Ouellet V, Zietarska M, Portelance L, Lafontaine J, Madore J, Puiffe ML, Arcand SL, Shen Z, Hebert J, Tonin PN, Provencher DM, Mes-Masson AM (2008). Characterization of three new serous epithelial ovarian cancer cell lines. BMC Cancer.

[12] Network TCGAR (2011). Integrated genomic analyses of ovarian carcinoma. Nature.

[13] Ahmed AA, Etemadmoghadam D, Temple J, Lynch AG, Riad M, Sharma R, Stewart C, Fereday S, Caldas C, Defazio A, Bowtell D, Brenton JD (2010). Driver mutations in TP53 are ubiquitous in high grade serous carcinoma of the ovary. J Pathol.

[14] McAlpine JN, Porter H, Kobel M, Nelson BH, Prentice LM, Kalloger SE, Senz J, Milne K, Ding J, Shah SP, Huntsman DG, Gilks CB (2012). BRCA1 and BRCA2 mutations correlate with TP53 abnormalities and presence of immune cell infiltrates in ovarian high-grade serous carcinoma. Mod Pathol.

[15] Press JZ, De Luca A, Boyd N, Young S, Troussard A, Ridge Y, Kaurah P, Kalloger SE, Blood KA, Smith M, Spellman PT, Wang Y, Miller DM, Horsman D, Faham M, Gilks CB (2008). Ovarian carcinomas with genetic and epigenetic BRCA1 loss have distinct molecular abnormalities. BMC Cancer.

[16] Bashashati A, Ha G, Tone A, Ding J, Prentice LM, Roth A, Rosner J, Shumansky K, Kalloger S, Senz J, Yang W, McConechy M, Melnyk N, Anglesio M, Luk MT, Tse K (2013). Distinct evolutionary trajectories of primary high-grade serous ovarian cancers revealed through spatial mutational profiling. J Pathol.

[17] Gorringe KL, George J, Anglesio MS, Ramakrishna M, Etemadmoghadam D, Cowin P, Sridhar A, Williams LH, Boyle SE, Yanaihara N, Okamoto A, Urashima M, Smyth GK, Campbell IG, Bowtell DD (2010). Copy number analysis identifies novel interactions between genomic loci in ovarian cancer. PLoS One.

[18] Wojnarowicz PM, Oros KK, Quinn MC, Arcand SL, Gambaro K, Madore J, Birch AH, de Ladurantaye M, Rahimi K, Provencher DM, Mes-Masson AM, Greenwood CM, Tonin PN (2012). The genomic landscape of TP53 and p53 annotated high grade ovarian serous carcinomas from a defined founder population associated with patient outcome. PLoS One.

[19] Alkema NG, Tomar T, van der Zee AG, Everts M, Meersma GJ, Hollema H, de Jong S, van Vugt MA, Wisman GB (2014). Checkpoint kinase 2 (Chk2) supports sensitivity to platinum-based treatment in high grade serous ovarian cancer. Gynecol Oncol.

[20] Keita M, Bachvarova M, Morin C, Plante M, Gregoire J, Renaud MC, Sebastianelli A, Trinh XB, Bachvarov D (2013). The RUNX1 transcription factor is expressed in serous epithelial ovarian carcinoma and contributes to cell proliferation, migration and invasion. Cell Cycle.

[21] Dong R, Liu X, Zhang Q, Jiang Z, Li Y, Wei Y, Li Y, Yang Q, Liu J, Wei JJ, Shao C, Liu Z, Kong B (2014). miR-14 inhibits tumor growth and metastasis by targeting metadherin in high-grade serous ovarian carcinoma. Oncotarget.

[22] Chapman-Rothe N, Curry E, Zeller C, Liber D, Stronach E, Gabra H, Ghaem-Maghami S, Brown R (2013). Chromatin H3K27me3/H3K4me3 histone marks define gene sets in high-grade serous ovarian cancer that distinguish malignant, tumour-sustaining and chemo-resistant ovarian tumour cells. Oncogene.

[23] Domcke S, Sinha R, Levine DA, Sander C, Schultz N (2013). Evaluating cell lines as tumour models by comparison of genomic profiles. Nat Commun.

[24] Jacob F, Nixdorf S, Hacker NF, Heinzelmann-Schwarz VA (2014). Reliable in vitro studies require appropriate ovarian cancer cell lines. J Ovarian Res.

[25] DelloRusso C, Welcsh PL, Wang W, Garcia RL, King MC, Swisher EM (2007). Functional characterization of a novel BRCA1-null ovarian cancer cell line in response to ionizing radiation. Mol Cancer Res.

[26] Sakai W, Swisher EM, Jacquemont C, Chandramohan KV, Couch FJ, Langdon SP, Wurz K, Higgins J, Villegas E, Taniguchi T (2009). Functional restoration of BRCA2 protein by secondary BRCA2 mutations in BRCA2-mutated ovarian carcinoma. Cancer Res.

[27] Letourneau IJ, Quinn MC, Wang LL, Portelance L, Caceres KY, Cyr L, Delvoye N, Meunier L, de Ladurantaye M, Shen Z, Arcand SL, Tonin PN, Provencher DM, Mes-Masson AM (2012). Derivation and characterization of matched cell lines from primary and recurrent serous ovarian cancer. BMC Cancer.

[28] Tonin PN, Mes-Masson AM, Futreal PA, Morgan K, Mahon M, Foulkes WD, Cole DE, Provencher D, Ghadirian P, Narod SA (1998). Founder BRCA1 and BRCA2 mutations in French Canadian breast and ovarian cancer families. Am J Hum Genet.

[29] Oros KK, Ghadirian P, Greenwood CM, Perret C, Shen Z, Paredes Y, Arcand SL, Mes-Masson AM, Narod SA, Foulkes WD, Provencher D, Tonin PN (2004). Significant proportion of breast and/or ovarian cancer families of French Canadian descent harbor 1 of 5 BRCA1 and BRCA2 mutations. Int J Cancer.

[30] Roy R, Chun J, Powell SN (2012). BRCA1 and BRCA2: different roles in a common pathway of genome protection. Nat Rev Cancer.

[31] Huen MS, Sy SM, Chen J (2010). BRCA1 and its toolbox for the maintenance of genome integrity. Nat Rev Mol Cell Biol.

[32] Kurman RJ (2013). Origin and molecular pathogenesis of ovarian high-grade serous carcinoma. Ann Oncol.

[33] Payne DJ, Gwynn MN, Holmes DJ, Pompliano DL (2007). Drugs for bad bugs: confronting the challenges of antibacterial discovery. Nat Rev Drug Discov.

[34] Singer G, Oldt R, Cohen Y, Wang BG, Sidransky D, Kurman RJ, Shih Ie M (2003). Mutations in BRAF and KRAS characterize the development of low-grade ovarian serous carcinoma. J Natl Cancer Inst.

[35] Wojnarowicz PM, Provencher DM, Mes-Masson AM, Tonin PN (2012). Chromosome 17q25 genes, RHBDF2 and CYGB, in ovarian cancer. Int J Oncol.

[36] Kobel M, Kalloger SE, Lee S, Duggan MA, Kelemen LE, Prentice L, Kalli KR, Fridley BL, Visscher DW, Keeney GL, Vierkant RA, Cunningham JM, Chow C, Ness RB, Moysich K, Edwards R (2013). Biomarker-based ovarian carcinoma typing: a histologic investigation in the ovarian tumor tissue analysis consortium. Cancer Epidemiol Biomarkers Prev.

[37] Kommoss S, Gilks CB, Kommoss F, Chow C, Hilpert F, du Bois A, Kobel M, Huntsman DG, Anglesio M, Kalloger SE, Pfisterer J (2013). Accelerating type-specific ovarian carcinoma research: Calculator for Ovarian Subtype Prediction (COSP) is a reliable high-throughput tool for case review. Histopathology.

[38] Laury AR, Perets R, Piao H, Krane JF, Barletta JA, French C, Chirieac LR, Lis R, Loda M, Hornick JL, Drapkin R, Hirsch MS (2011). A comprehensive analysis of PAX8 expression in human epithelial tumors. Am J Surg Pathol.

[39] Madore J, Ren F, Filali-Mouhim A, Sanchez L, Kobel M, Tonin PN, Huntsman D, Provencher DM, Mes-Masson AM (2010). Characterization of the molecular differences between ovarian endometrioid carcinoma and ovarian serous carcinoma. J Pathol.

[40] Meden H, Kuhn W (1997). Overexpression of the oncogene c-erbB-2 (HER2/neu) in ovarian cancer: a new prognostic factor. Eur J Obstet Gynecol Reprod Biol.

[41] Serrano-Olvera A, Duenas-Gonzalez A, Gallardo-Rincon D, Candelaria M, De la Garza-Salazar J (2006). Prognostic, predictive and therapeutic implications of HER2 in invasive epithelial ovarian cancer. Cancer Treat Rev.

[42] Provencher DM, Lounis H, Champoux L, Tetrault M, Manderson EN, Wang JC, Eydoux P, Savoie R, Tonin PN, Mes-Masson AM (2000). Characterization of four novel epithelial ovarian cancer cell lines. In Vitro Cell Dev Biol Anim.

[43] el-Deiry WS, Kern SE, Pietenpol JA, Kinzler KW, Vogelstein B (1992). Definition of a consensus binding site for p53. Nat Genet.

[44] el-Deiry WS (1998). Regulation of p53 downstream genes. Semin Cancer Biol.

[45] Kato S, Han SY, Liu W, Otsuka K, Shibata H, Kanamaru R, Ishioka C (2003). Understanding the function-structure and function-mutation relationships of p53 tumor suppressor protein by high-resolution missense mutation analysis. Proc Natl Acad Sci U S A.

[46] Lee MK, Teoh WW, Phang BH, Tong WM, Wang ZQ, Sabapathy K (2012). Cell-type, dose, and mutation-type specificity dictate mutant p53 functions in vivo. Cancer Cell.

[47] Zietarska M, Maugard CM, Filali-Mouhim A, Alam-Fahmy M, Tonin PN, Provencher DM, Mes-Masson AM (2007). Molecular description of a 3D in vitro model for the study of epithelial ovarian cancer (EOC). Mol Carcinog.

[48] Meunier L, Puiffe ML, Le Page C, Filali-Mouhim A, Chevrette M, Tonin PN, Provencher DM, Mes-Masson AM (2010). Effect of ovarian cancer ascites on cell migration and gene expression in an epithelial ovarian cancer in vitro model. Transl Oncol.

[49] Fujita M, Enomoto T, Murata Y (2003). Genetic alterations in ovarian carcinoma: with specific reference to histological subtypes. Mol Cell Endocrinol.

[50] Kobel M, Huntsman D, Gilks CB (2008). Critical molecular abnormalities in high-grade serous carcinoma of the ovary. Expert Rev Mol Med.

[51] Do K, Chen AP (2013). Molecular pathways: targeting PARP in cancer treatment. Clin Cancer Res.

[52] Gelmon KA, Tischkowitz M, Mackay H, Swenerton K, Robidoux A, Tonkin K, Hirte H, Huntsman D, Clemons M, Gilks B, Yerushalmi R, Macpherson E, Carmichael J, Oza A (2011). Olaparib in patients with recurrent high-grade serous or poorly differentiated ovarian carcinoma or triple-negative breast cancer: a phase 2, multicentre, open-label, non-randomised study. Lancet Oncol.

[53] Ledermann J, Harter P, Gourley C, Friedlander M, Vergote I, Rustin G, Scott C, Meier W, Shapira-Frommer R, Safra T, Matei D, Macpherson E, Watkins C, Carmichael J, Matulonis U (2012). Olaparib maintenance therapy in platinum-sensitive relapsed ovarian cancer. N Engl J Med.

[54] Liu JF, Konstantinopoulos PA, Matulonis UA (2014). PARP inhibitors in ovarian cancer: current status and future promise. Gynecol Oncol.

[55] (2015). Olaparib Approved for Advanced Ovarian Cancer. Cancer Discov.

[56] Cavallone L, Arcand SL, Maugard CM, Nolet S, Gaboury LA, Mes-Masson AM, Ghadirian P, Provencher D, Tonin PN (2010). Comprehensive BRCA1 and BRCA2 mutation analyses and review of French Canadian families with at least three cases of breast cancer. Fam Cancer.

[57] Tonin PN, Maugard CM, Perret C, Mes-Masson AM, Provencher DM (2007). A review of histopathological subtypes of ovarian cancer in BRCA-related French Canadian cancer families. Fam Cancer.

[58] Kreuzinger C, Gamperl M, Wolf A, Heinze G, Geroldinger A, Lambrechts D, Boeckx B, Smeets D, Horvat R, Aust S, Hamilton G, Zeillinger R, Cacsire Castillo-Tong D (2015). Molecular characterization of 7 new established cell lines from high grade serous ovarian cancer. Cancer Lett.

[59] Langdon SP, Lawrie SS, Hay FG, Hawkes MM, McDonald A, Hayward IP, Schol DJ, Hilgers J, Leonard RC, Smyth JF (1988). Characterization and properties of nine human ovarian adenocarcinoma cell lines. Cancer Res.

[60] Kalluri R, Weinberg RA (2009). The basics of epithelial-mesenchymal transition. J Clin Invest.

[61] Stewart CJ, McCluggage WG (2013). Epithelial-mesenchymal transition in carcinomas of the female genital tract. Histopathology.

[62] Auer K, Bachmayr-Heyda A, Aust S, Sukhbaatar N, Reiner AT, Grimm C, Horvat R, Zeillinger R, Pils D (2015). Peritoneal tumor spread in serous ovarian cancer-epithelial mesenchymal status and outcome. Oncotarget.

[63] Vousden KH, Prives C (2009). Blinded by the Light: The Growing Complexity of p53. Cell.

[64] Brady CA, Attardi LD (2010). p53 at a glance. J Cell Sci.

[65] Aylon Y, Oren M (2011). New plays in the p53 theater. Curr Opin Genet Dev.

[66] Vousden KH, Lu X (2002). Live or let die: the cell's response to p53. Nat Rev Cancer.

[67] Vousden KH, Ryan KM (2009). p53 and metabolism. Nat Rev Cancer.

[68] Mahmoudi S, Henriksson S, Corcoran M, Mendez-Vidal C, Wiman KG, Farnebo M (2009). Wrap53, a natural p53 antisense transcript required for p53 induction upon DNA damage. Mol Cell.

[69] Vilborg A, Wilhelm MT, Wiman KG (2010). Regulation of tumor suppressor p53 at the RNA level. J Mol Med (Berl).

[70] Saldana-Meyer R, Recillas-Targa F (2011). Transcriptional and epigenetic regulation of the p53 tumor suppressor gene. Epigenetics.

[71] Yemelyanova A, Vang R, Kshirsagar M, Lu D, Marks MA, Shih Ie M, Kurman RJ (2011). Immunohistochemical staining patterns of p53 can serve as a surrogate marker for TP53 mutations in ovarian carcinoma: an immunohistochemical and nucleotide sequencing analysis. Mod Pathol.

[72] Birch AH, Arcand SL, Oros KK, Rahimi K, Watters AK, Provencher D, Greenwood CM, Mes-Masson AM, Tonin PN (2011). Chromosome 3 anomalies investigated by genome wide SNP analysis of benign, low malignant potential and low grade ovarian serous tumours. PLoS One.

[73] Sereni MI, Baldelli E, Gambara G, Zanotti L, Bandiera E (2015). Functional characterization of epithelial ovarian cancer histotypes by drug target-based protein signaling activation mapping: implications for personalized cancer therapy. Proteomics.

[74] Zannoni GF, Morassi F, Prisco MG, De Stefano I, Vellone VG, Arena V, Scambia G, Gallo D (2012). Clinicopathologic and immunohistochemical features of ovarian clear cell carcinomas in comparison with type I and type II tumors. Int J Gynecol Pathol.

[75] Heintz AP, Odicino F, Maisonneuve P, Quinn MA, Benedet JL, Creasman WT, Ngan HY, Pecorelli S, Beller U (2006). Carcinoma of the ovary. FIGO 26th Annual Report on the Results of Treatment in Gynecological Cancer. Int J Gynaecol Obstet.

[76] Lounis H, Provencher D, Godbout C, Fink D, Milot MJ, Mes-Masson AM (1994). Primary cultures of normal and tumoral human ovarian epithelium: a powerful tool for basic molecular studies. Exp Cell Res.

[77] Belanger MH, Dolman L, Arcand SL, Shen Z, Chong G, Mes-Masson AM, Provencher D, Tonin PN (2015). A targeted analysis identifies a high frequency of BRCA1 and BRCA2 mutation carriers in women with ovarian cancer from a founder population. J Ovarian Res.

[78] Oros KK, Arcand SL, Bayani J, Squire JA, Mes-Masson AM, Tonin PN, Greenwood CM (2013). Analysis of genomic abnormalities in tumors: a review of available methods for Illumina two-color SNP genotyping and evaluation of performance. Cancer Genet.

[79] Puiffe ML, Le Page C, Filali-Mouhim A, Zietarska M, Ouellet V, Tonin PN, Chevrette M, Provencher DM, Mes-Masson AM (2007). Characterization of ovarian cancer ascites on cell invasion, proliferation, spheroid formation, and gene expression in an in vitro model of epithelial ovarian cancer. Neoplasia.

[80] Franken NA, Rodermond HM, Stap J, Haveman J, van Bree C (2006). Clonogenic assay of cells in vitro. Nat Protoc.

