# Supplementary material for: Novel high-grade serous epithelial ovarian cancer cell lines that reflect the molecular diversity of both the sporadic and hereditary disease
Source: Genes Cancer. 2015 Sep;6(9-10):378–98. doi: 10.18632/genesandcancer.76 (PMC4633166; doi:10.18632/genesandcancer.76)
Supplement: Supplementary file 1 [file ganc-06-378-s001.pdf]

Novel high-grade serous epithelial ovarian cancer cell lines that reflect the molecular diversity of both the sporadic and hereditary disease

Supplementary Material

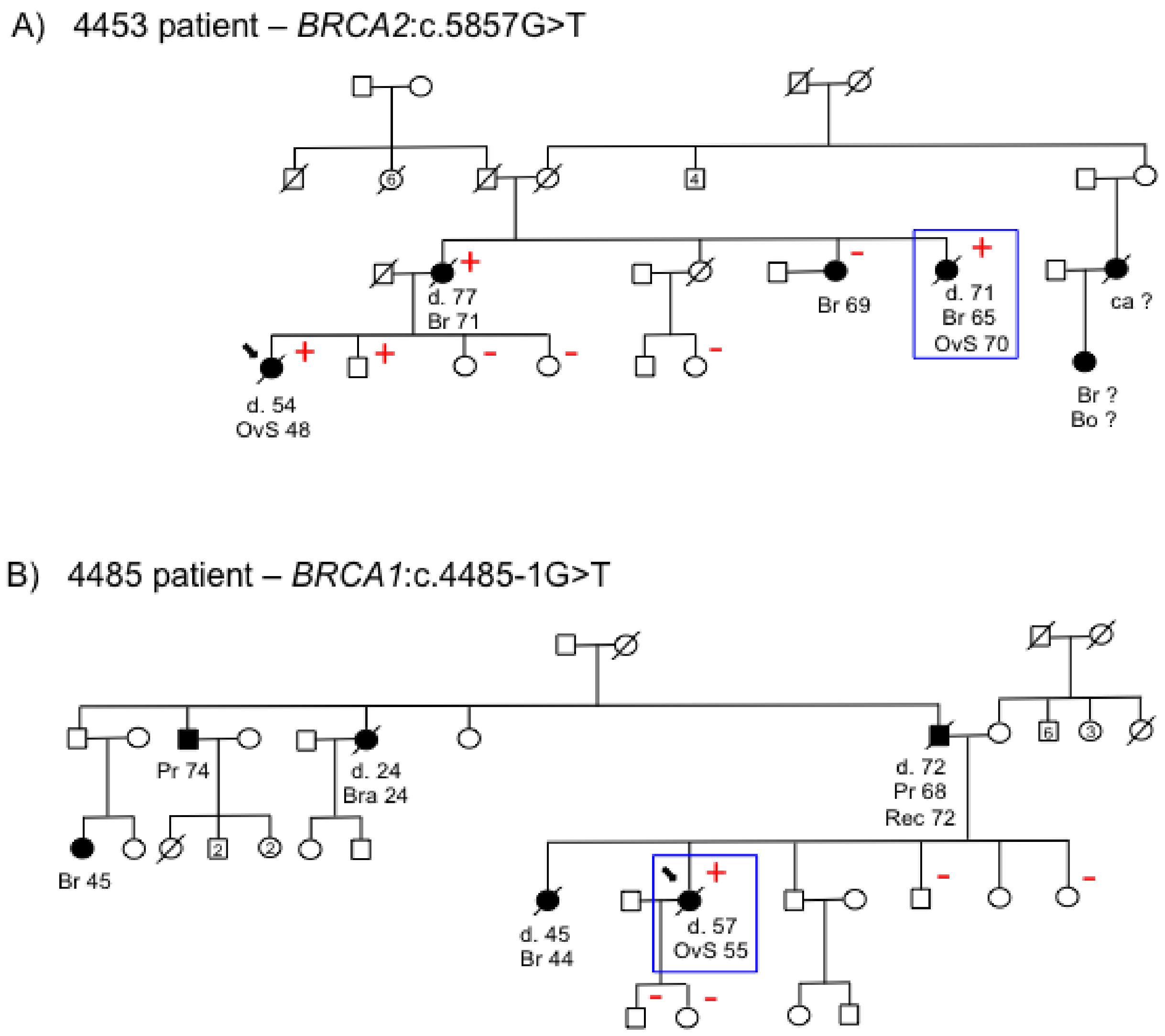

Supplemental Figure S1 – Pedigrees of *BRCA2*:c.5857G>T (E1953X) (patient 4453) and *BRCA1*:c.4485-1G>T (splice) (patient 4485) mutation carrier families. Shown are truncated pedigreeas of mutation carrier families from the ovarian cancer patients 4453 and 4485 (blue rectangles) from which the cell lines were derived. An arrow indicates the proband; and mutation carrier status is denoted by plus or minus red sign for tested family members. Note that three other family members from the 4453 patient also harbor the *BRCA2* mutation. On the other hand, patient 4485 is the only mutation carrier in the family. Ages at death (d.) are indicated if known along with ages at diagnosis of cancer. Abbreviations: breast cancer (Br), serous ovarian cancer (OvS), bone cancer (Bo), prostate cancer (Pr), brain tumor (Bra), and unknown cancer type (ca).

Supplemental Table S1 – IHC conditions for staining of paraffin-embedded formalin-fixed EOC tissue samples.

	Retrieval		Primary antibody	
**Marker**	**Cell Conditioning**	**Incubation time (min)**	**Dilution**	**Incubation time (min)**
p53	#2	60	1/200	32
WT1	#1	60	1/200	44
PAX8	#1	36	1/300	32
HER2	#1	60	1/650	60
CK7	#1	60	1/200	44
CK8	#1	60	1/100	60
CK18	#1	30	1/1000	40
CK19	#1	60	1/2000	40
